# Patient involvement in clinical trials

**DOI:** 10.1038/s43856-022-00156-x

**Published:** 2022-07-25

**Authors:** Jan Geißler, Emily Isham, Gary Hickey, Clive Ballard, Anne Corbett, Cheryl Lubbert

**Affiliations:** 1 The Patvocates Network, Munich, Germany; 2 Rare Cancers Australia, Bowral, NSW Australia; 3 National Institute for Health Research, University of Southampton, Southampton, UK; 4 School of Healthcare Enterprise & Innovation, University of Southampton, Southampton, UK; 5 University of Exeter Medical School, College of Medicine & Health, University of Exeter, Exeter, UK; 6 Reverba, Seattle, WA USA

**Keywords:** Clinical trials

## Abstract

Clinical trials are required to ensure new treatments are safe and effective for patients. The involvement of participants in the planning and execution of clinical trials is critical not only to their success but also so that the participating communities can benefit from them.

In this Viewpoint, people who have been involved in clinical trials in a variety of roles discuss how clinical trial participants can be involved in clinical trials and how their involvement can maximise the success of clinical trials and improve the patient experience.

## Jan Geißler

**Figure:**
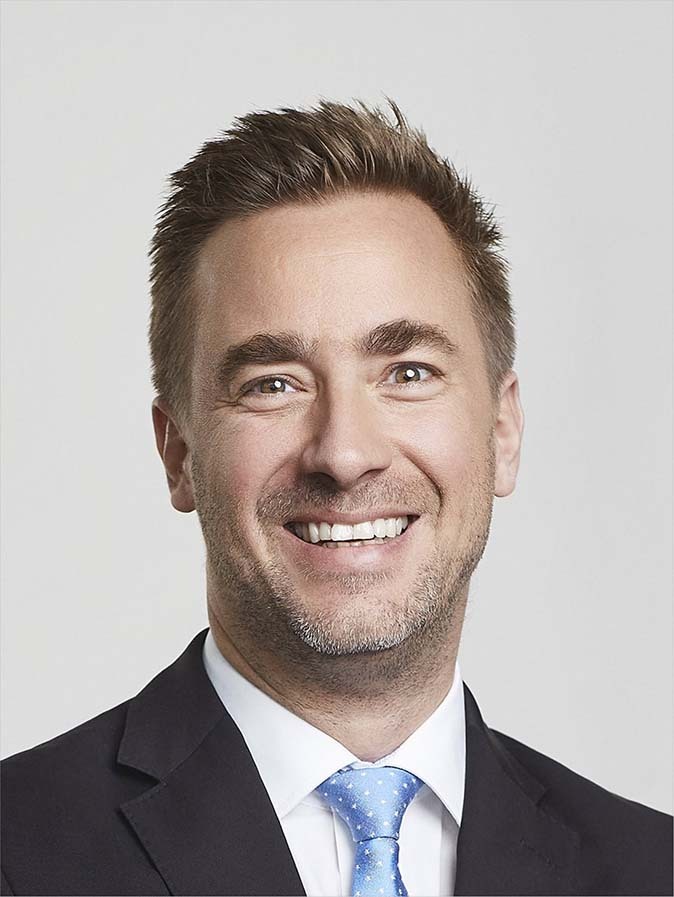
© the Author

I was diagnosed with chronic myeloid leukaemia in 2001, at the age of 28, and successfully treated in an early phase clinical trial. Soon after, I decided to commit my professional life to advocating on behalf of rare cancer patients in Germany and Europe. I have founded several international patient organisations, and served on committees of medical societies, the European Commission, and medical journals. I particularly seek to improve patient outcomes through initiatives and projects of my think tank, Patvocates, which is dedicated to patient advocacy and patient involvement in research.

Patients need faster and more innovative research and can contribute significantly to clinical trials that are designed to truly meet their unmet needs and priorities, if they are involved as partners in the research process at an early stage. However, patient involvement in clinical trials regularly takes place too late in the research process, if at all. Hence, clinical trials are often solely focused on solving clinically relevant challenges and meeting endpoints. They should focus on addressing the priorities, preferences and needs of those whose lives are on the line, or have a compromised quality of life through symptom or therapy burden^1^.

In view of the fact that patients make themselves available for these clinical trials, research conducted without patient involvement in the design may address less meaningful research questions and waste resources on studies which patients do not want to join^2^. It also raises strong ethical questions, even more so if quality of life data are not collected, or if patient-relevant results, especially negative outcomes, are not being published. This may lead to duplication of research, and to therapeutic choices which are focused on clinical rather than patient-relevant outcomes.

Patient advocates have in-depth insights into what living with a disease and therapy means to patients, and are experts in the needs of patient communities and their subgroups. They can share their experience and evidence on patient preferences, patient-relevant outcomes, and outcomes in a real-world setting. There are many aspects that need to be taken into account from the patients’ perspective when research is being designed. These include weighing up expected risks and benefits, the burden of the disease and adverse events in everyday life, such as its influence on, for example, ability to work, social life and mental well-being. Both short- and long-term consequences of disease and therapy need to be considered. It is too late to do so when trial data are being analysed.

The patient involvement roadmap of the European Patients’ Academy (EUPATI)^3^ defines multiple points at which patients as trained experts and advisors can actively participate in the drug development process. For instance, they can be involved in the definition of research questions and patient-relevant endpoints, trial design and conduct, inclusion and exclusion criteria and patient priorities in terms of benefit and risk. They can contribute to the selection of meaningful instruments to measure patient-reported adverse events, patient-reported outcomes and quality of life. And they can contribute to the analysis of evidence, and to the dissemination of research by writing informed consent documents, patient reviews and lay summaries in patient-friendly language. Patients can also act as reviewers and co-authors of peer-reviewed articles^4^.

Recently, patient involvement in research has also become a political goal, strengthened by the joint declaration of the German, Portuguese and Slovenian Trio Presidency of the Council of the EU in September 2021 on “Principles of Successful Patient Engagement in Cancer Research”^5^. The paper, which represents a consensus of more than 130 experts from various stakeholder groups in 16 countries, declares that patients should actively be involved in research design from the outset, and calls for a cultural change in science toward more patient participation. This change would benefit all stakeholders in clinical trials. The patient community’s motto “Nothing about us without us” has never been more tangible than today.

## Emily Isham

**Figure:**
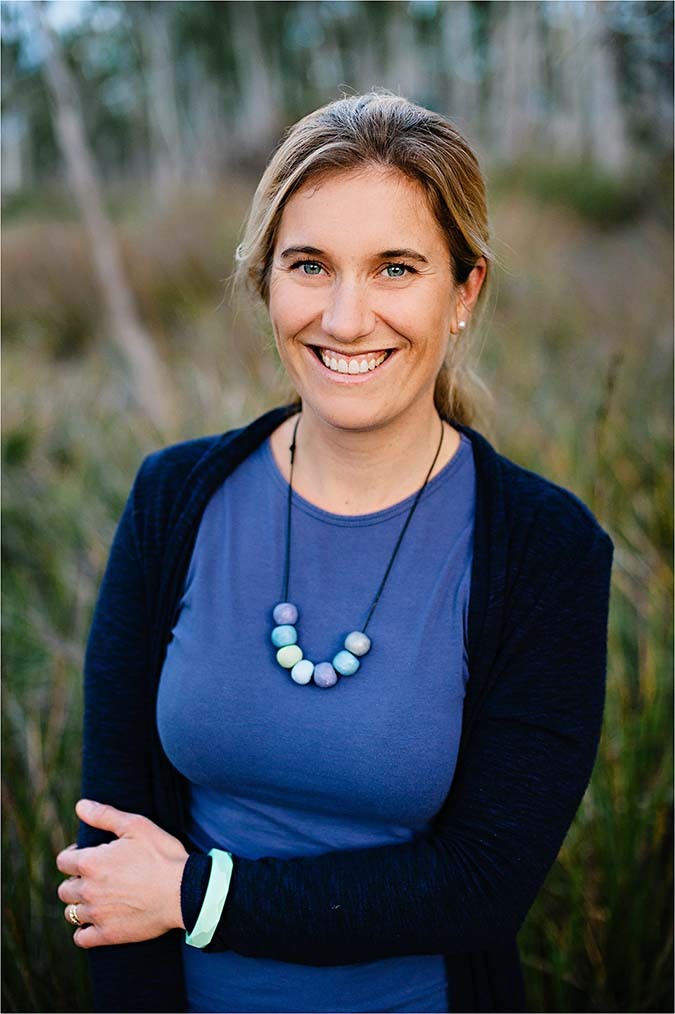
Lisa Kuilenburg, © the Author

At the age of two my son was unexpectedly diagnosed with cancer. As parents, we were confronted with the horror of our child’s life-threatening diagnosis. We felt grief for the future—the stolen childhoods of our children, the ill health of our son and the reduced number of years of ordinary family life.

For a patient with cancer, time marches on, with no capacity for pause while awaiting for emerging treatments to jump through all the hoops of approval and funding. As Rare Cancers Australia CEO, Richard Vines, often says “Treatment delayed is treatment denied”^6^. Sometimes, it can feel that this ‘hoop-jumping’ obscures the views of those people and families suffering at the centre.

These days, I often hear the phrase ‘patient-centred care’. The initial phrase was conceived by Balint when trying to convey the need for each person to be understood within the context of their unique needs^7^, rather than focusing strictly on the disease. It has caused a profound cultural shift from the previously paternalistic model of medicine, conveying the idea of healthcare being individualised, taking particular circumstances and values into account, rather than being blanket plans dictated by medical professionals or institutions. The phrase is the epitome of where healthcare is going, as medical technology becomes more sophisticated, and specific treatments are being developed for an individual’s cancer subtype. At a deeper level, patient-centred care involves engaging with patients more holistically, respecting their need to be active collaborators and fully-informed decision makers. This can be even more complex when the patient is a child, in which case family-centred care captures the broader dynamics at play, assuming that the parents are making decisions in their child’s best interests.

So are we there yet? Patients are often involved in clinical trials as a part of their care pathway. There is evidence that those who partake in clinical trials as part of their cancer treatment do better than others with the same cancer^8^. However, unfortunately, my experience enroling my son in a clinical trial suggests attention to patient-centricity is not yet present as the standard in all trials. I felt that my son as a patient was viewed more as a data source, rather than as a key player, as has been described by others^9^.

In addition, the necessity for local clinical trial research and enrolment is particularly significant for my family, after the six of us had to abruptly leave our home, school and workplaces for our son’s cancer treatment. Our 6-year-old Ned had been on harsh treatment for relapsed high-risk pre-B acute lymphoblastic leukaemia for 4 years, including a bone marrow transplant from his 2-year-old sister, before we had to urgently travel to Seattle for his enrolment on a CAR-T clinical trial. This was an established, evidence-based treatment showing good results, and yet it was not available in Australia at the time. On top of being uprooted when at our most vulnerable, we were thrust into a new healthcare system, hospital, and medical team. Preparations for the treatment pathways ahead were fraught and unsupported, and the only reason I was somewhat involved with pre-trial discussions was because of my medical training—my non-medically trained husband was unable to properly participate. We were vulnerable, uncertain, and felt at the mercy of numerous treating teams spread worldwide. Many other patients are in the position that they cannot relocate in order to access alternative treatments^10^.

So how could this experience have been different? How do we overhaul this current model and incorporate patients at the centre? How do we embrace autonomy and non-maleficence when gathering data from patients as they undergo often-gruelling treatment^10^? Patients should feel empowered and supported in making decisions—being allowed to ask questions, to bring support people to appointments, to have options explained, and to be given time to make their own choices, respecting their own psychological, physical and social needs where possible. Patients should also be told about the various available clinical trials, with remote participation an option whenever possible^11^. It should also be made clear when patients opinions can be considered, and explanations given when they cannot be acted on.

For a disease so consequential as cancer, it is imperative that patients and their caregivers are all in the room when options are considered and decisions are made—more so when the only options are clinical trials. When someone’s life hangs in the balance, we should be asking how we can make that person feel heard and confident in the treatment decisions they make.

## Gary Hickey

**Figure:**
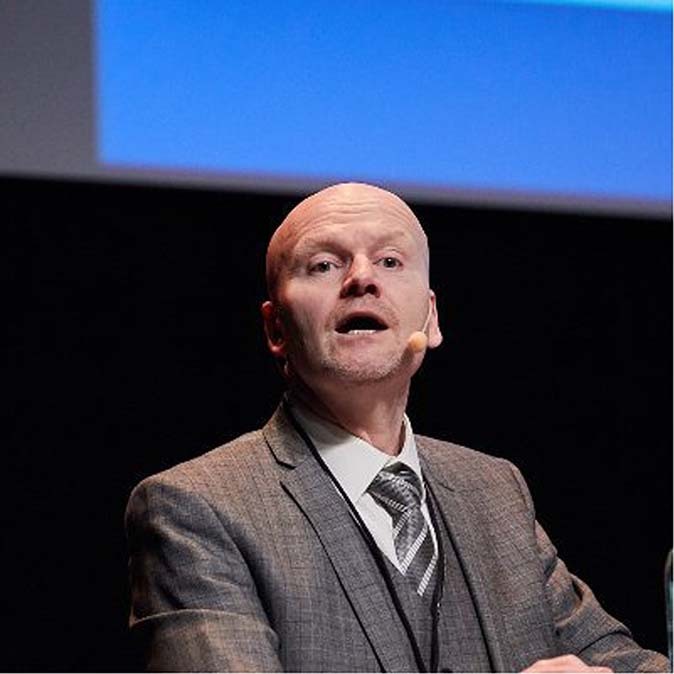
© the Author

I am both a researcher, involving patients and the public in research, and a provider of advice and guidance on patient and public involvement in research. Currently, I am developing an online course on patient and public involvement and engagement in research, targeted at members of the public and researchers less experienced or new to patient and public involvement and engagement. Patient and public involvement in clinical trials is important for two key reasons. Firstly, patients and the public can improve the relevance and quality of the research. Patients and the public have lived experience of a condition or service and so their expertise and knowledge are different, but just as valuable, as that which researchers and clinicians bring to a study. For example, patients and the public will have greater insight into the impact of a treatment or service on them and their quality of life. Secondly, there is the moral imperative that people should be involved in research that is likely to have an impact on them. Furthermore, research funders are increasingly making it a mandatory requirement in research proposals.

Patient and public involvement can take place in every stage of the research cycle, from the development of proposals through to the dissemination of the research. In some clinical trials, much of the patient and public involvement tends to take place in the design and development of recruitment materials. For example, ensuring that Participant Information Sheets and Consent Forms are legible and in plain language, and advising researchers on where to go to recruit people into their studies; indeed, sometimes they can provide access to the relevant group. Patients and the public also often advise on the design of clinical trials; on how best to construct the design to ensure optimum comfort and suitability for trial participants. They can also help shape the research questions, research instruments and outcome measures ensuring that the research is of relevance to end users (i.e. patients and the public as well as researchers and clinicians) and that questionnaires and interview questions are comprehensive and legible to patients and public.

However, successful patient and public involvement won’t just happen without effort. It needs resourcing in terms of finance and support. Patients and the public need payment for their time and expertise. They may need support to enable them to contribute; for example, training on how clinical trials work and/or pastoral support as they negotiate their way through the unfamiliar research process. And, of course, researchers and clinicians may also need training and support in how to engage and communicate with patients and the public. Crucially, all parties need to move away from any notion that one type of expertise is superior to that of another; they are just different. But collectively they can result in improved research and better outcomes.

## Clive Ballard

**Figure:**
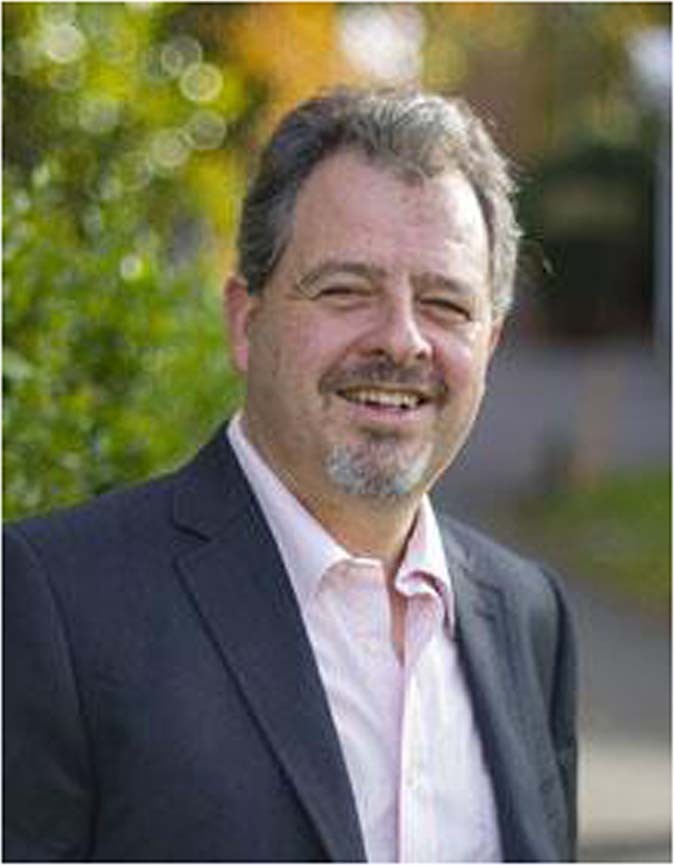
© the Author

## Anne Corbett

**Figure:**
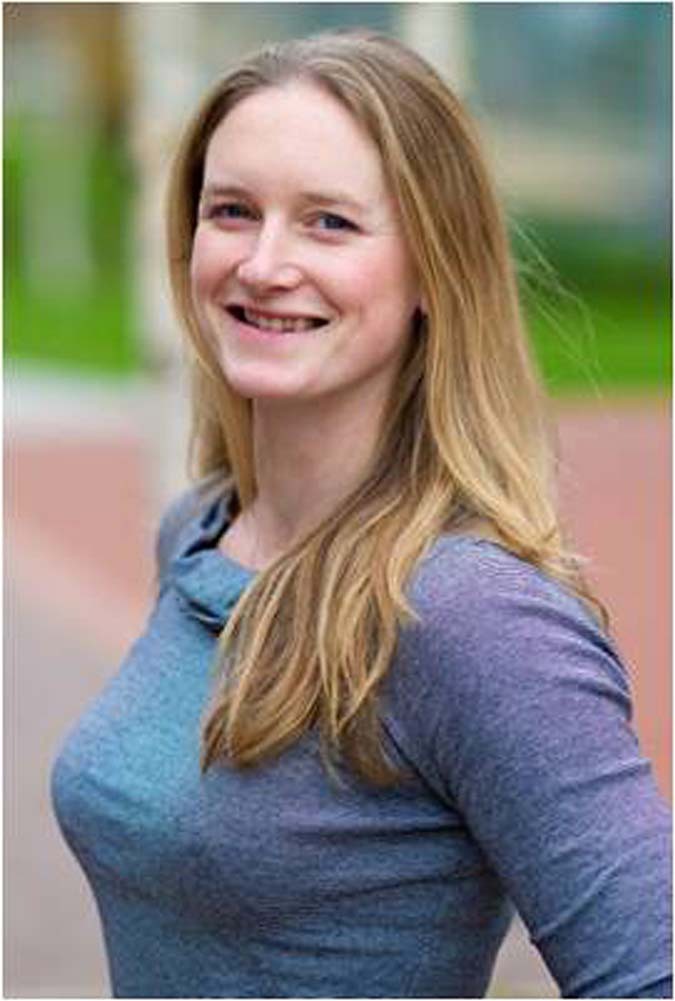
© the Author

The increasing emphasis on patient involvement in clinical trial design is welcome and extremely positive. We deliver a portfolio of clinical trials in the cognition and dementia space, with a particular focus on complex, multicentre trials of both pharmacological and psychosocial interventions in people with dementia. We also have experience in leading online trials including nutraceutical and digital interventions in older adults.

From our perspective, one extremely valuable aspect of patient involvement in clinical trials is to ensure that trial outcomes are relevant to individuals, particularly in fields where outcomes are complex to interpret. An example is in trials of antipsychotic medications for the treatment of neuropsychiatric symptoms in dementia. This is a challenging condition to treat, with subjective differences in symptoms, high risk of distress and lack of mental capacity in patients, which prevents them from providing consent and communicating their needs and wishes. Outcome measures for these symptoms often show poor correlation with quality of life measures and lack a tangible impact on people’s lives. Involvement of patients and their families have helped to enable more meaningful outcome measures for these trials, such as tailored quality of life and selection of focused neuropsychiatric symptoms scales. A second example is in trials of cholinesterase inhibitor treatments such as donepezil and memantine, where modest benefits in performance on neuropsychological assessments have not captured the equally important improvements that patients experience in their day-to-day functioning. These are often related to broader benefits, such as improved confidence, which may be captured through less clinically-focused measures such as Goal Attainment Scaling, in which patient involvement has been key in identifying the most meaningful outcomes, and the best way of supporting people to identify and monitor their personal goals

Of equal importance is the discussion of risk tolerability and the borders of ethical acceptance. Discussions often arise around what level of risk is acceptable. This may differ depending on the stage and certainty of a person’s diagnosis, or on the likelihood of a treatment being effective. A novel, first-in-class treatment may be considered riskier than a modified existing therapy, and this may define how a trial can be designed. Patients can contribute valuably to discussions on risks and benefits of particular treatments.

More recently, we have recognised the value of patient input into trials conducted in digital environments where participants take part remotely. This is a relatively new approach and we have gained considerable insight from participant feedback which has shaped recruitment and retention strategies and ensured safe participation of patients contributing from home without direct clinical supervision.

The benefits of patient involvement are not without their challenges. Diversity and inclusion is a major factor. Historically, there has been a tendency to recruit a small number of patient advisors, with a bias towards white, well-educated and financially secure individuals. It is imperative to move towards larger, broader, more inclusive groups to capture the full range of views, concerns and aspirations. A specific dilemma within our field occurs when patients do not have the capacity to make informed decisions. Determining the best approach to capture the patient voice under those circumstances is challenging but vital. It is key that the academic community takes ownership of these challenges and takes a proactive stance in understanding and applying patient perspectives to improve trial design.

## Cheryl Lubbert

**Figure:**
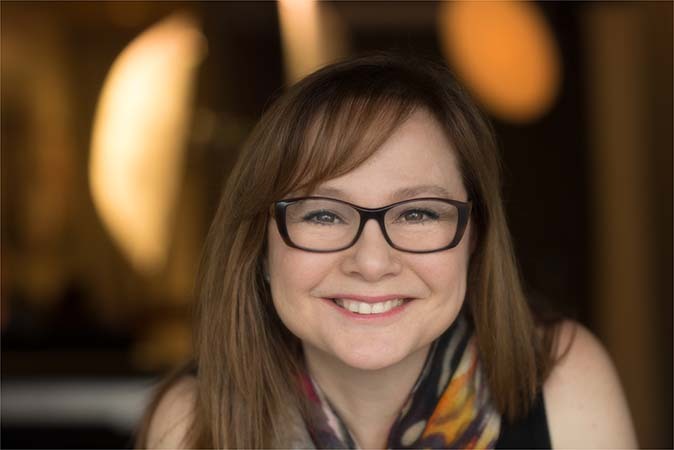
© the Author

I have more than 25 years of senior management experience at Fortune 500 companies, including Bristol-Myers Squibb, Immunex, Inc., Amgen and Abbott Labs, where I built and led the company’s Immunology franchise, including the Humira (adalimumab) business, helping to create the foundation for this blockbuster global brand. I was an executive member of the Immunology clinical trial planning committee at Abbott Labs that was responsible for strategic planning and approval for clinical trials including Phase 2–4 for new compounds, diseases, indications and REMS (Risk Evaluation and Mitigation Strategy). Additionally, I was involved in the new indication trial planning and protocol design at Amgen as well as strategic planning for Phase 4 trials for Enbrel.

I am now CEO and co-founder of Reverba, a global patient engagement company that has been creating transformative tech-enabled solutions to build a bridge between patients and biopharma companies, from clinical development through commercialisation, for more than 20 years.

The rise of smart technologies such as smartphones with app-enabled health monitors, disease-specific social media forums and virtual healthcare provider platforms have fundamentally changed the role patients play in all aspects of their own healthcare, from how they gather information to how and when they relate to others with their condition and their medical team. Providing what patients need, where they want to access it, in language that is easy to understand, is now more than just a trend—it is an expectation. And this expectation particularly applies to clinical trials. Excluding patients from the clinical trial process can cost both time and budget. According to a Tufts University study, the cost for changes made during a clinical trial can range from $141,000–$500,000+ per change. Clinical trial recruiter Beroe reports that once underway, 85% of clinical trials fail to retain enough patients to reach completion^12^.

Patients want to play an active role in clinical trials. In a survey we conducted through Reverba’s subsidiary Health Stories Project, we asked 352 patients about their clinical trial knowledge and experience. Among respondents, 88% were familiar with clinical trials, but 65% had not participated in a trial. When asked, 97% of patients said it was important for biopharma to include consumer input when designing trials, and 83% were interested in providing feedback.

It is critical to include patients not only in clinical trial design, but with ongoing input throughout the clinical trial cycle. Before the start, patients can provide feedback to improve protocol design and improve recruitment. Feedback during the trial enables the clinical trial experience to be improved and the trial to be adapted as needed. After the trial, patients can be involved in post-trial results communication to build relationships.

There are many ways to engage with patients to incorporate their input as you plan a clinical trial. Patient advisory boards bring together a small group of patients and caregivers (6–10) either in person or virtually to share perspectives on the trial. One-on-one interviews with patients provide in-depth conversations, which can guide decision making that considers the patient perspective across a range of patients. A patient consultant takes on a more active role and becomes a member of the clinical trial team, participating in multiple meetings and calls during the trial planning process.

With engaged and active patients, their involvement should not end when the trial begins. During the trial, an in-trial insights platform can be used to conduct surveys or interviews with patients currently enroled for real-time feedback. After the trial, a clinical trial alumni programme enables companies to maintain communication with patients to provide information about the study results and whether a product is approved.

Patients provide real-world insight that can help support better trials across a company’s therapeutic areas. Pharma companies can also take a more agile approach, incorporating what they learn from patients to impact how trials are designed in the future—faster, more efficient, more cost-effective and providing a better experience for participants.

Thus, engaging health consumers throughout the clinical trial process has the potential to help get drugs to market faster and truly change healthcare.
